# Do carbon nanoparticles really improve thyroid cancer surgery? A retrospective analysis of real-world data

**DOI:** 10.1186/s12957-020-01852-5

**Published:** 2020-05-02

**Authors:** Junsong Liu, Chongwen Xu, Rui Wang, Peng Han, Qian Zhao, Honghui Li, Yanxia Bai, Lifeng Liu, Shaoqiang Zhang, Xiaobao Yao

**Affiliations:** 1 Department of Otorhinolaryngology-Head and Neck Surgery, The First Affiliated Hospital of Xi’an Jiaotong University, Clinical Research Center for Thyroid Diseases of Shaanxi Province, 277 West Yanta Road, Xi’an, 710061 Shaanxi, People’s Republic of China; 2 Department of Anesthesiology, The First Affiliated Hospital of Xi’an Jiaotong University, 277 West Yanta Road, Xi’an, 710061 Shaanxi, People’s Republic of China

**Keywords:** Carbon nanoparticles, Thyroidectomy, Parathyroid, Lymph node dissection

## Abstract

**Background:**

Parathyroid protection and central neck dissection (CND) are basic points of thyroid cancer surgery and draw persistent concern. We aimed to evaluate the value of carbon nanoparticles (CNs) for parathyroid gland protection and CND in thyroid surgery for thyroid cancer patients.

**Methods:**

A total of 386 consecutive thyroid cancer patients were enrolled in the retrospective study. Three hundred thirty-four patients using CNs intraoperatively were included in the CN group, and 52 patients without using CNs or any other helping agent were included in the control group. Intact parathyroid hormone (iPTH) was examined. Medical records and histopathologic reports were reviewed. Histopathologic examination was performed.

**Results:**

There were no statistical significances in demographic and basic surgical information, preoperative iPTH, and serum calcium between the two groups (*P* > 0.05). In the CN group, the thyroid tissue and central neck lymph nodes were stained black by CNs, while the parathyroid glands were not. Histopathological examination showed that the carbon nanoparticles might accumulated in the subcapsular sinus of lymph nodes compared with the none-stained samples. The staining with CNs did not impact the histopathological examination. There were no significant differences in postoperative hypocalcemia and hypoPT at day 1, 1 month, and half year after surgery between the two groups, respectively. There was a big decline of iPTH level after surgery, whereas the perioperative decreasing amplitude of PTH was not statistically different between the CNs and control group (57.2 ± 28.6 vs 55.7 ± 27.8, *P* = 0.710). There were 43 patients occurring incidental parathyroidectomy in the CN group (43/334, 12.9%) and 7 patients in the control group (7/52, 13.5%), without significant difference (*P* = 0.907). There was no significant difference in the number of lymph nodes identified by pathology per patient between the CNs and control group regardless of unilateral and bilateral CND.

**Conclusions:**

Carbon nanoparticles help highlight parathyroid glands and lymph nodes in thyroidectomy, but generate no significant benefit for parathyroid glands protection and lymph node dissection. The value of carbon nanoparticles in thyroid cancer surgery should not be exaggerated and needs further evaluation.

## Background

With the rapid increasing of thyroid cancer patients, thyroidectomy plus central neck dissection (CND) is more and more performed by endocrine or head and neck surgeons [1, 2]. During the operation, the protection of parathyroid glands and the radical dissection of lymph nodes in the central neck compartment are commonly concerned issues which affect the patients’ quality of life and prognosis after surgery [3, 4]. The accurate identification of parathyroid glands is needed in order to protect parathyroid gland function [5]. Techniques for parathyroid identification draw broad concerns.

**Fig. 1:**
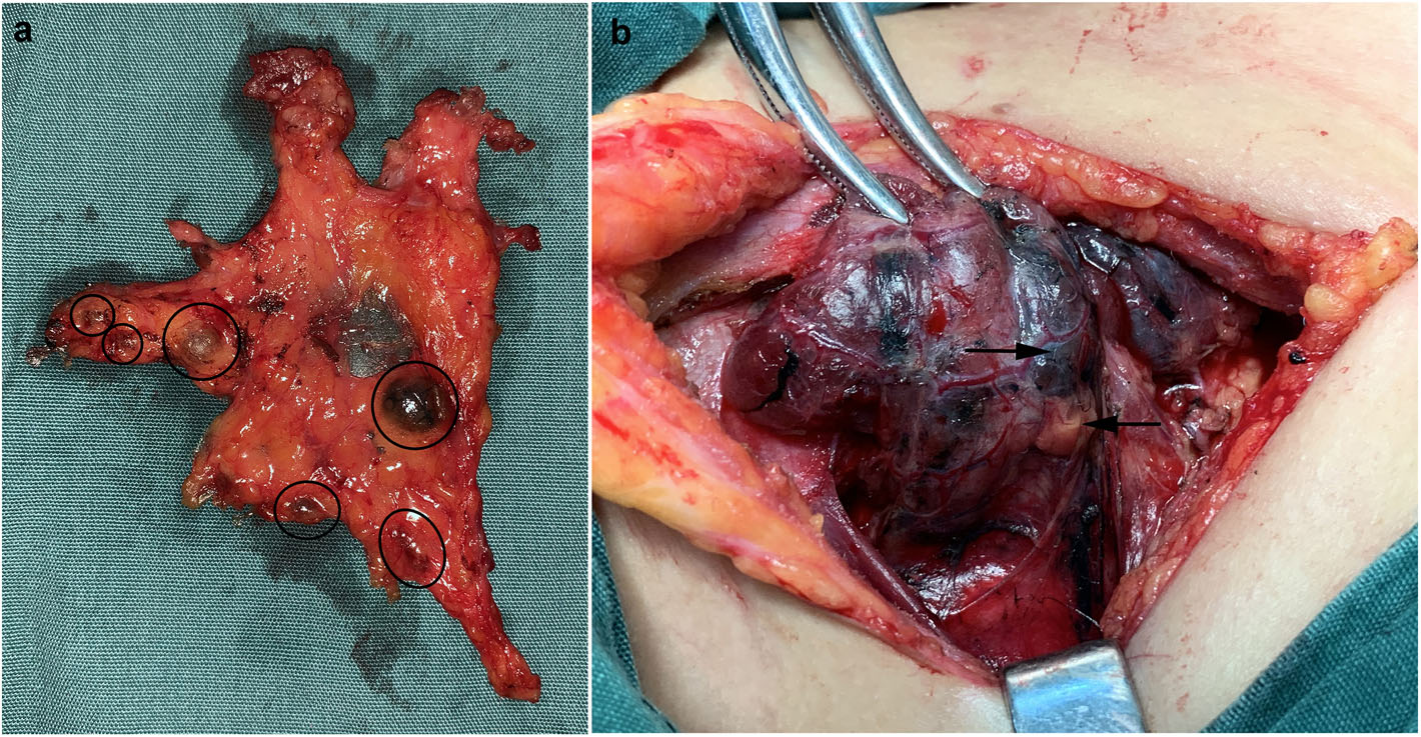
**a** Staining of lymph nodes by carbon nanoparticles. The specimen of central neck dissection shows that the lymph nodes at the drainage area of the thyroid lymph were stained black by the carbon nanoparticles. The circles indicate CNs stained lymph nodes. **b** The thyroid gland was stained black by carbon nanoparticles, while the parathyroid gland was not. Carbon nanoparticle suspension was injected into normal thyroid tissue at two or three spots randomized and uniformed distributed at the upper, median, and lower part of thyroid lobe. Then, thyroid tissue was stained black but the parathyroid glands were not because of sharing different lymphatic system from thyroid. →indicates black stained thyroid tissue, ←indicates parathyroid gland

Carbon nanoparticle (CN) suspension (China Food and Drug Administration approval H20041829, Chongqing Lummy Pharmaceutical Co., Ltd., Chongqing, China) is a lymphatic tracing agent originally used in the operations of breast and gastrointestinal cancers [6, 7]. In recent years, CNs have been widely used in thyroid surgeries in China [8]. The diameter of the particle is 150 nm, so it can enter the lymphatic capillaries (the intercellular space of lymphatic capillary cells is 120–500 nm), but not the blood capillaries (the intercellular space of blood capillary cells is 20–50 nm). When injected into thyroid tissue, CNs would stain the drainage lymph node black (Fig. 1a) and guide lymph node dissection [8]. Additionally, as the parathyroid glands share different lymphatic system from thyroid, the parathyroid glands are not stained by CNs (Fig. 1b). Therefore, CNs can be used for parathyroid gland identification and provide protection for parathyroid glands in thyroidectomies [9]. Recent clinical studies and systemic reviews showed that CNs helped protect parathyroid glands, decrease unintentional removal of parathyroid glands, and therefore decrease postoperative hypocalcemia and hypoparathyroidism (hypoPT) [10, 11]. CNs also increased the number of lymph nodes identified by pathology [12]. However, several studies found that CNs did not improve long-term clinical results [13, 14]. And queries about the value of CNs in thyroid surgery still exist [15, 16], especially among those experienced surgeons in tertiary hospitals.

In the present study, we conducted a retrospective cohort study to evaluate the value of CNs for parathyroid gland protection and lymph node dissection through the analysis of a series of patients undergoing total thyroidectomy plus central neck lymph node dissection.

## Materials and methods

### Materials

Carbon nanoparticle (CN) suspension is a lymphatic tracing agent (China FDA approval H20041829, Chongqing Lummy Pharmaceutical Co., Ltd., Chongqing, China). Up to now, it is a safe agent without side effects reported [12].

### Patients

The study was approved by the Institutional Review Board of The First Affiliated Hospital of Medical College of Xi’an Jiaotong University, Xi’an, Shaanxi, China. Informed written consent was obtained from the enrolled patients. Patients diagnosed with thyroid cancer and undergone total thyroidectomy with CND from November 2017 to October 2018 at the Department of Otorhinolaryngology-Head and Neck Surgery, The First Affiliated Hospital of Xi’an Jiaotong University (Xi’an, Shaanxi, China) were enrolled retrospectively. The inclusion criterion was primary treated thyroid cancer. Exclusion criteria included patients with benign thyroid tumor, past medical history of cervical surgery or radiotherapy, and previously diagnosed parathyroid dysfunction. The carbon nanoparticles suspension is 1-mL specification and the price is about 2400 yuan RMB, not covered in the basic medical insurance. Therefore, not all the patients chose to use CNs. Patients were included in the CN group or control group according to the usage of CNs or not.

### Surgical procedure and CN suspension injection

All the operations were performed by professional and experienced head and neck surgeon group. Total thyroidectomy plus unilateral or bilateral CND was performed following the standard procedure. A 1-mL syringe was used for CN suspension injecting into the thyroid gland. Three to five injecting spots were randomized and uniformed distributed at the upper, median, and lower part of each thyroid lobe. The suspension was injected into normal thyroid tissue, beneath thyroid capsule, but not into the blood vessel or thyroid cancer tissue. The total volume of CN suspension injected into thyroid was 0.1–0.3 mL per patient. Appropriate manual pressure was applied on each injecting spot to prevent solution leakage to the surrounding operation field. Five minutes was needed for particle diffusion before subsequent procedures. Then, we could see the CNs gradually diffused in the thyroid tissue, from the injecting spots to the surrounding area, until the entire thyroid lobe was uniformly stained black. Later, we could also find some lymph nodes stained black at central compartment of the neck (Fig. 1a). On the contrary, the parathyroid glands were not stained because of sharing different lymphatic systems (Fig. 1b).

### Perioperative management

Serum calcium and intact parathyroid hormone (iPTH) were routinely tested preoperatively and at day 1 postoperatively. When patients complained of symptoms of calcium deficiency, oral supplement of calcium tablets (0.25 μg bid P.O.) and vitamin D (600 mg bid P.O.) were applied for patients to alleviate hypocalcemia symptoms. Intravenous calcium was given if necessary.

### Data collection and histopathological examination

Data were retrospectively collected from medical records of the hospital information system. All patients were followed up for at least 6 months through outpatient visits. Data at 1 month and 6 months after operation were collected. The diameter of primary tumor was obtained from preoperative neck ultrasound report. Operation records and histopathologic reports were reviewed for the extent of surgery and to evaluate incidental parathyroidectomy, the number of lymph nodes identified, and thyroid sizes. Histopathologic evaluation was performed by experienced pathologists of The Department Pathology. Hypoparathyroidism (hypoPT) was defined according to the American Thyroid Association Statement on Postoperative Hypoparathyroidism [17]. Permanent hypoPT is defined when serum PTH is less than the lower limit of the hospital-specific reference range and continues beyond 6 months after surgery. The lower limit of intact PTH at the hospital standard is 15 pg/mL. ∆PTH = (PTH before surgery–PTH of day 1 after surgery)/PTH before surgery × 100%.

### Statistical analysis

Continuous variables were presented as mean ± SD. Statistical analysis was performed using the Student *t* test, chi-square test, or Mann-Whitney *U* test according to data distribution characteristics through the SPSS 17.0 software (SPSS Inc., Chicago, IL, United States). *P* value less than 0.05 was considered statistically significant.

## Results

### General information

A total of 386 consecutive thyroid cancer patients were enrolled in the retrospective study. Of them, 334 patients using CNs intraoperatively were included in the CN group, and 52 patients without using CNs or any other helping agent were included in the control group for analysis. The general information and basic clinical data of the two groups were shown in Table 1. There were no significant differences in gender, age, tumor size, thyroid size, range of CND, concomitant comorbidity, concomitant thyroiditis or hyperthyroidism, preoperative PTH, and serum calcium between the two groups (*P* > 0.05). There were 93 and 14 patients with concomitant comorbidities in the CNs and control group, respectively. The most common concomitant comorbidity was hypertension (68 cases, 74.2% vs 11 cases, 78.6%) in the CNs and control group, without statistical difference in distribution (*P* > 0.05).

**Table 1:** General information and basic data of the two groups

	CN group (*n* = 334)	Control group (*n* = 52)	*χ*^2^(*t/Z*) value	*P* value
Gender (*n*, %)				
Male	86 (25.7%)	12 (23.1%)	*χ*^2^ = 0.170	0.681
Female	248 (74.3%)	40 (76.9%)		
Age (ss, years)	44.0 ± 11.7	46.6 ± 13.0	*t* = − 1.48	0.139
Thyroid size ( $\overline{x}\pm s$,, cm)*	4.5 ± 0.9	4.7 ± 1.1	*Z* = − 1.064	0.287
Tumor size (*n*, %)				
T > 4 cm	7 (2.1%)	4 (7.7%)	*χ*^2^ = 5.746	0.125
2 cm < T ≤ 4 cm	62 (18.6%)	11 (21.2%)		
1 cm < T ≤ 2 cm	131 (39.2%)	20 (38.5%)		
T ≤ 1 cm	134 (40.1%)	17 (32.7%)		
Range of CND (*n*, %)				
Bilateral CND	115 (34.4%)	19 (36.5%)	*χ*^2^ = 0.071	0.789
Unilateral CND	217 (65.0%)	33 (63.5%)		
Without CND	2 (0.6%)	0 (0%)		
Comorbidity (*n*, %)				
No	241 (72.2%)	38 (73.1%)	*χ*^2^ = 0.019	0.890
Yes	93 (27.8%)	14 (26.9%)		
Accompanied by thyroiditis or hyperthyroidism (*n*, %)				
No	256 (76.6%)	39 (75%)	*χ*^2^ = 0.068	0.795
Yes	78 (23.4%)	13 (25%)		
Preoperative PTH ($\overline{x}\pm s$, pg/mL)	46.6 ± 18.1	46.3 ± 16.9	*Z* = − 0.12	0.905
Preoperative serum calcium ($\overline{x}\pm s$,, mmol/L)	2.24 ± 0.12	2.23 ± 0.12	*t* = 0.038	0.969

*CNs* carbon nanoparticles, *CND* central neck dissection, *PTH* parathyroid hormone

*Thyroid size represents the longitudinal length of the excisional thyroid lobe on main lesion side

CNs helped highlight parathyroid glands but did not prevent postoperative iPTH decline or decrease postoperative hypocalcemia and hypoPT in total thyroidectomy.

In clinical practice, CNs did help highlight parathyroid glands. The thyroid tissue was stained black while the parathyroid tissue was not (Fig. 1b). Table 2 presented the incidences of postoperative hypoPT at different time points. There were no significant differences in hypocalcemia and hypoPT at day 1, 1 month, and half year postoperatively between the two groups, respectively. There was a big decline of iPTH level after surgery. But the perioperative decreasing amplitude of PTH was not statistically different between the CN group and control group ((57.2 ± 28.6)% vs (55.7 ± 27.8)%, *P* = 0.710). The results suggested that CNs did help highlight parathyroid glands, but did not decrease both transient and permanent postoperative hypocalcemia and hypoPT in total thyroidectomy for thyroid cancer patients.

**Table 2:** Comparison of postoperative PTH and serum calcium in CNs and control groups

Results	CN group	Control group	*χ*^2^(*t/Z*) value	*P* value
**Day one after surgery**				
PTH (*n*, %)				
≥ 15 pg/mL	177/326 (54.3%)	31/51 (60.8%)	*χ*^2^ = 0.751	0.386
< 15 pg/mL	149/326 (45.7%)	20/51 (39.2%)		
∆PTH(%, $\overline{x}\pm s$,)	(57.2 ± 28.6)%	(55.7 ± 27.8)%	*Z* = − 0.372	0.710
Serum calcium (*n*, %)				
≥ 2.1 mmol/L	142/325 (43.7%)	19/50 (38.0%)	*χ*^2^ = 0.573	0.449
< 2.1 mmol/L	183/325 (56.3%)	31/50 (62.0%)		
**1 month after surgery**				
PTH (*n*, %)				
≥ 15 pg/mL	251/271 (92.6%)	43/45 (95.6%)	*χ*^2^ = 0.160	0.689
< 15 pg/mL	20/271 (7.4%)	2/45 (4.4%)		
Serum calcium (*n*, %)				
≥ 2.1 mmol/L	275/312 (88.1%)	32/40 (80.0%)	*χ*^2^ = 2.107	0.147
< 2.1 mmol/L	37/312 (11.9%)	8/40 (20.0%)		
**Half year after surgery**				
PTH (*n*, %)				
≥ 15 pg/mL	300/311 (96.5%)	49/50 (98.0%)	*χ*^2^ = 0.019	0.890
< 15 pg/mL	11/311 (3.5%)	1/50 (2.0%)		
Serum calcium (*n*, %)				
≥ 2.1 mmol/L	291/314 (92.7%)	42/47 (89.4%)	*χ*^2^ = 0.250	0.617
< 2.1 mmol/L	23/314 (7.3%)	5/47 (10.6%)		
**Unintentional parathyroid removal** (*n*, %)				
No	291/334 (87.1%)	45/52 (86.5%)	*χ*^2^ = 0.014	0.907
Yes	43/334 (12.9%)	7/52 (13.5%)		

*CNs* carbon nanoparticles, *PTH* parathyroid hormone, ∆PTH = (PTH before surgery–PTH of day 1 after surgery)/PTH before surgery × 100%

CNs did not decrease incidental parathyroidectomy in thyroidectomy.

Through the review of postoperative histopathologic reports, we evaluated the incidence of unintentional removal of parathyroid glands. There were 43 patients found incidental parathyroidectomy in the CN group (43/334, 12.9%) and 7 patients in the control group (7/52, 13.5%). The data did not show significant difference between the two groups (*P* = 0.907; Table 2). In majority of the cases (49/50) with incidental parathyroidectomy, one parathyroid gland was removed. In one patient of the CN group, two parathyroid glands were unintentionally dissected. The data indicated that the use of CNs did not decrease incidental removal of parathyroid.

CNs highlighted locoregional lymph nodes but did not improve central neck lymph node dissection in thyroid cancer patients.

**Fig. 2:**
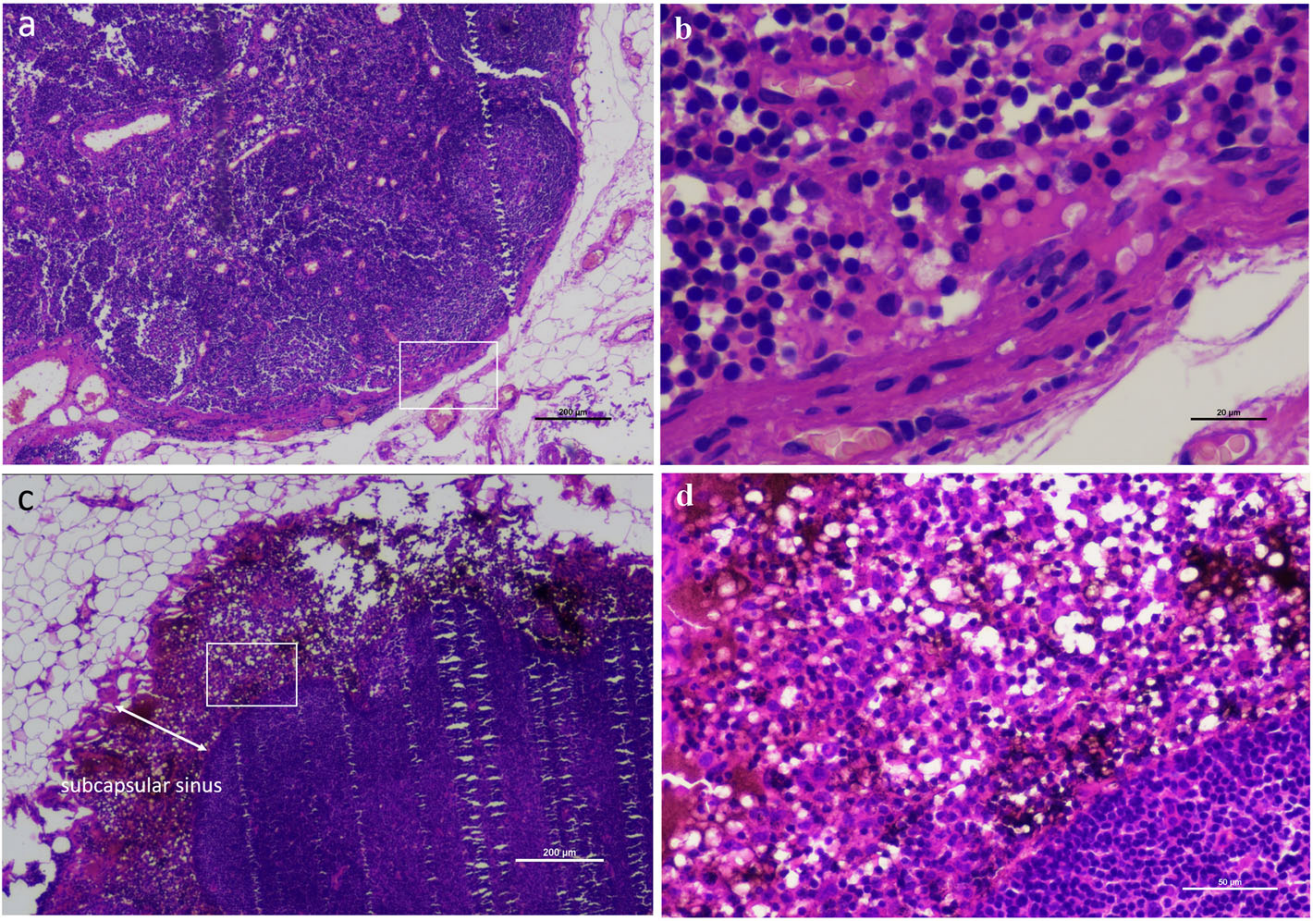
**a** and **b** Normal lymph node section without carbon nanoparticle staining. As a control sample, there was no sign of abnormal staining in the lymph node sections. **c** and **d** Lymph node section showed carbon nanoparticle staining. Histopathological examination showed that the carbon nanoparticles (the dark brown to black staining area) accumulated in the subcapsular sinus of lymph nodes in the case using carbon nanoparticles suspension. ↔indicates the subcapsular sinus area of a lymph node. Figure magnification: **a** × 40, **b** × 400, **c** × 40, and **d** × 200. **b** Shows the square frame area of **a**. **d** Shows the square frame area of **c**

In the cases using CNs, the locoregional lymph nodes around thyroid gland were stained black (Fig. 1a). Histopathological examination showed that the carbon nanoparticles accumulated in the subcapsular sinus of lymph nodes compared with the none-stained samples (Fig. 2). The particles did not enter the medulla of lymph node or metastatic carcinoma cells; thus, the staining with CNs did not impact the histopathological examination. Among the enrolled patients, 65.0% of the CN group and 63.5% of the control group underwent unilateral CND, and 34.4% of the CN group and 36.5% of the control group underwent bilateral CND, respectively (Table 1). For the patients undergoing bilateral CND, the number of lymph nodes identified by pathology per patient was 9.81 ± 5.68 and 11.06 ± 7.84 in the CN group and control group, respectively, and it was not statistically different (*P* = 0.859; Table 3). For the cases with unilateral CND, there was no statistical difference in the number of lymph nodes identified by pathology per patient between the CN group and control group (5.88 ± 4.12 vs 6.02 ± 4.44, *P* = 0.830; Table 3), either. The rate of central neck lymph node metastasis was slightly higher in the CN group (176/334, 52.7%) than the control group (24/52, 46.2%), but without statistical significance (*P* = 0.380). These data suggested that the use of CNs may highlight locoregional lymph nodes but did not improve central neck lymph node dissection or affect lymph node metastasis evaluation in thyroid cancer patients.

**Table 3:** Comparison of the number of lymph nodes identified by pathology and central neck lymph node metastasis in the CNs and control groups

	CN group (*n* = 334)	Control group (*n* = 52)	*χ*^2^(*t/Z*) value	*P* value
Number of identified lymph nodes ($\overline{x}\pm s$)				
Unilateral CND	5.88 ± 4.12	6.02 ± 4.44	*t* = − 0.215	0.830
Bilateral CND	9.81 ± 5.68	11.06 ± 7.84	*Z* = − 0.178	0.859
Central neck lymph node metastasis (*n*/*N*, %)				
LN (+)	176/334 (52.7%)	24/52 (46.2%)	*χ*^2^ = 0.771	0.380
LN (−)	158/334 (47.3%)	28/52 (53.8%)		

*CNs* carbon nanoparticles, *CND* central neck dissection, *LN* lymph node

## Discussion

Total thyroidectomy plus central neck lymph node dissection for thyroid cancer patients is a frequently performed operation. The most common complication of the surgery is postoperative hypoparathyroidism because of injury or unintentional removal of parathyroid glands [18, 19]. It can lead to numbness in the hands and feet, even tetany, and thus negatively affect patients’ quality of life, even cause fatal convulsions, cardiac arrhythmia, heart failure, and chronic renal failure. Data showed that the incidence of inadvertent removal of parathyroid glands during thyroidectomy ranged 8–19%, depending on the surgeon’s experience and the type of surgical procedures [20]. The incidence of permanent hypocalcemia after total thyroidectomy is about 0–3.8% [21]. So, it is vitally important to protect parathyroid glands in thyroidectomy. Parathyroid developing techniques drew broad concerns.

Nanomedicine uses nanoparticles for therapeutic, diagnostic, and preventative purposes and has achieved promising results in various aspects of medical applications, mainly in cancer therapy [22]. Duo to their specific properties in size, shape, and chemical composition, nanoparticles presented promising capabilities by surface modification. Drug delivery systems constructed from nanoparticles showed good biocompatibility, hemocompatibility, penetrating, and targeting ability, and thus improved drug efficacy and reduced toxicity [23–25]. Some anticancer drugs have obtained approval for clinical use, such as liposomal paclitaxel [26] and Marqibo (vincristine sulfate liposome injection) [27]. Other applications include functionalized nanoprobes for fluorescent bioimaging [28], nanomaterial-enabled radiosensitization [29], and lymphatic tracing agent [7].

In recent years, carbon nanoparticle suspension has been widely used and reported in Chinese clinical practice for its potential to improve parathyroid protection and guide lymph node dissection in thyroid surgeries. A recent meta-analysis showed that, compared with the blank control group, the use of CNs increased the number of retrieved lymph nodes by 3.39 per patient, decreased the rate of accidental parathyroid removal, transient, and permanent hypoparathyroidism by approximately 22%, 31%, and 24%, respectively [10]. But there was no statistical difference in postoperative permanent hypocalcemia rate between the CNs and control group. In another study, Xue found that the use of CNs just decreased the incidence of transient hypoparathyroidism and hypocalcemia at the 2nd and 5th day after surgery, but did not decrease permanent hypoparathyroidism and hypocalcemia [14]. CNs did not reduce cancer recurrence after at least 5-year follow-up. Therefore, they advised not to exaggerate the function of CNs because it could not improve long-term clinical results. In the present study, CNs did not reduce the incidence of postoperative hypocalcemia and hypoPT, and incidental removal of parathyroid glands. The overall parathyroid protection results in our study were comparable to previous reports from the perspectives of declining iPTH [13], the rate of incidental parathyroid removal [10], and the rate of permanent hypothyroidism [21], respectively. But carbon nanoparticles did not improve clinical results in thyroid surgery.

For experienced and skilled endocrine surgeons in tertiary hospitals, majority of parathyroid glands can be well protected even without the help of imaging agent. In this situation, the use of CNs may not provide practical help in parathyroid protection. In the present study, the incidence of postoperative hypocalcemia, hypoPT, and incidental removal of parathyroid glands, even without using CNs, were comparable to the previous studies. So, CNs may provide help for unexperienced surgeons, but do not value much for those with abundant experiences. Additionally, parathyroid protection requires not only accurate identification and in situ preserving, but also careful protection of its blood supply and venous drainage [30, 31]. In the present study, the thyroid tissue was stained black after CN injection while the parathyroid gland was not. CNs did help highlight parathyroid glands. But this did not improve clinical results. Thus, we suppose that CNs could not help protect parathyroid blood vessels. Even in some cases, the vestigial carbon nanoparticles might stain the operating field and add difficulty in protecting the arteries that supply parathyroid glands [13, 14]. Additionally, the vestigial carbon nanoparticles might induce immune response in the thyroid bed and increase tissue inflammation and edema, which would decrease the venous flow of parathyroid glands [13]. These factors might negatively affect the function of parathyroid glands and need further research and confirmation.

Another thing requires attention is that some surgeons use CNs to help distinguish parathyroid glands and lymph nodes according to whether the tissue is black stained. However, not all the lymph nodes in the drainage area could be stained black because of several reasons, such as the blockage of lymphatic flow and sinus and the interruption of lymphatic duct drainage [12]. Therefore, it is not precise enough to distinguish parathyroid glands from lymph nodes through the use of CNs.

As for CND, CNs did not increase the number of lymph nodes identified by pathology in the present study. The average number of lymph nodes identified by pathology in our study was no less than the previous reports regardless of the use of CNs [10, 11]. According to the guidelines and clinical procedures, the central compartment neck dissection was en bloc dissection of all the tissue (fat, fascia, and lymph node) in the defined space (ranging vertically from the hyoid bone to the innominate artery level, horizontally between the borders of bilateral carotid arteries) [32]. So, the number of harvested lymph nodes should not vary regardless of the use of CNs. Although in some studies, the black staining may increase the number of lymph nodes identified by pathology, but it did not affect long-term cancer recurrence rate [14]. Another thing should be taken into account is that the researchers might devote more effort to pick out the lymph nodes in the dissected fibrofatty tissue in prospective randomized controlled trials (RCTs). This may increase some lymph nodes that may not be identified in daily clinical work. This may thus lead to different results between RCTs and real-world retrospective data. Therefore, we suppose that CNs can highlight the lymph nodes and may have the potential to help detecting more lymph nodes, especially the small ones in specimens, but it will not affect the clinical result as long as you perform standard radical lymph node dissection.

Up to now, there have been no side effects reported on carbon nanoparticle suspension in previous studies [12]. As discussed above, the potential immune response induced by vestigial carbon nanoparticles in the thyroid bed might increase tissue inflammation and edema, which would decrease the venous flow of parathyroid glands and negatively affect the function of parathyroid glands [13]. But this needs further research and confirmation. Other risks include skin staining which can be avoided if carefully administered during surgery [14]. The carbon nanoparticles might have accumulated in the subcapsular sinus of lymph nodes could not enter the thyroid parenchymal cells or metastatic carcinoma cells; therefore, the staining with CNs did not impact histopathological examination.

Based on the available evidence, up to now, there are no studies performed on this carbon nanoparticle suspension in other countries or areas except in China. We did not get the exact reasons for this. Perhaps more efforts are needed to make it approved by FDA of other countries. Despites this, nanomedicine has drown worldwide concerns and achieved promising results in various aspects of medical applications, especially in cancer therapy. We believe that more and more concerns and studies will be focused on nanomedicine, including this carbon nanoparticle suspension.

## Conclusion

In conclusion, the study indicated that carbon nanoparticles helped highlight parathyroid glands and lymph nodes in thyroidectomy, but did not improve clinical results. The value of CNs in parathyroid gland protection and lymph node dissection in thyroid surgery should not be exaggerated, especially for experienced and skilled endocrine surgeons in tertiary hospitals. More real-world data from multiple centers may be needed for further evaluation.

## Data Availability

All data generated or analyzed during this study are included in this published article.

## Abbreviations

CNs: Carbon nanoparticles
CND: Central neck dissection
iPTH: Intact parathyroid hormone
